# The patient journey for people with dementia and their carers in Peru: From first symptoms to diagnosis and treatment

**DOI:** 10.1002/alz.71581

**Published:** 2026-07-09

**Authors:** Daniela Rossini‐Vilchez, Francisco Jose Tateishi‐Serruto, Christopher Butler, María Sofía Cuba‐Fuentes, Silvana Perez‐Leon, Miriam Lúcar‐Flores, Guillermo Almeida‐Huanca, L. Carolina Olaya‐Silva, J. Jaime Miranda, Maria Kathia Cardenas, Jose Carlos Vera Tudela, Antonio Bernabe‐Ortiz, Rafael A. Calvo, Francisco Diez‐Canseco, Filipa Trigo Landeiro, Graham Moore, Lee White, William Whiteley, María Lazo‐Porras, Jemma Hawkins, the IMPACT Salud Research Group

**Affiliations:** ^1^ CRONICAS Center of Excellence in Chronic Diseases Universidad Peruana Cayetano Heredia Lima Peru; ^2^ Department of Brain Sciences Imperial College London London UK; ^3^ The George Institute for Global Health UK London UK; ^4^ Center for Research in Primary Health Care Universidad Peruana Cayetano Heredia Lima Peru; ^5^ Health Economics Research Centre University of Oxford Oxford UK; ^6^ Sydney School of Public Health Faculty of Medicine and Health The University of Sydney Sydney New South Wales Australia; ^7^ Dyson School of Design Engineering Imperial College London London UK; ^8^ Centre for Development Evaluation Complexity and Implementation in Public Health Improvement (DECIPHer) School of Social Sciences Cardiff University Cardiff UK; ^9^ Centre for Clinical Brain Sciences University of Edinburgh Edinburgh UK

**Keywords:** caregiver, comorbidities, comorbidity, dementia, diagnosis, fragmented health system, patient journey, Peru, quality of care

## Abstract

**INTRODUCTION:**

We explored how people with dementia (PwD) and their carers navigate Peru's health system for diagnosis and treatment.

**METHODS:**

We interviewed PwD (*n* = 4), carers (*n* = 18), and healthcare workers (*n* = 17) from four sites. We developed a patient journey map using thematic analysis and participant validation.

**RESULTS:**

Three journey stages emerged. Pre‐diagnosis: Families seek care across different sub‐health systems and facilities 2–4 years after symptom onset. Diagnosis: Specialist consultation is sought in major cities and diagnosis occurs in hospitals based on clinical presentation. Continuity of care: Care focuses on controlling behavioral and psychological symptoms, with limited access to dementia‐specific drugs and non‐pharmacological interventions, and heavy reliance on carers’ resources. Community mental health centers (CMHCs) are preferred facilities due to geographical and specialist accessibility and perceived care quality.

**DISCUSSION:**

Without a formal entry point to dementia care, families navigate fragmented pathways, whereas CMHCs emerge as facilities with adequate care procedures for PwD.

## Highlights

- We developed an empirically grounded map that visualizes the journey of people with dementia (PwD) in the pursuit of diagnosis and care through the Peruvian Health System.
- We conducted in‐depth qualitative interviews with PwD, carers, and healthcare workers across four diverse sites in Peru to explore experiences of receiving diagnosis and treatment for dementia.
- Waiting times ranged from 1 to 10 years between the initial onset of symptoms and accessing a consultation at a health facility. Across the patient journey, emotions of carers function as forces that organize their actions and decisions.
- There is not a singular, coherent care pathway for dementia attention in the Peruvian health system. Instead, PwD and their families have a burden of responsibility to navigate a variety of routes, encountering diverse points of contact across levels of attention and systems, shaped by their resources.
- The main barriers to dementia diagnosis and treatment were a lack of trained specialists, medication shortages, and long waiting times for attention. The kind and respectful treatment provided by healthcare workers was recognized as a key facilitator, especially at the primary care level.

## 1 BACKGROUND

Dementia is an increasing public health priority worldwide, particularly in low‐ and middle‐income countries (LMICs), where population aging has amplified its impact. In the Americas, more than 10 million people are known to be living with dementia, and this number is expected to double every 20 years.^1^ In Peru, population‐based studies in urban settings reported a dementia prevalence of 9.34% in adults ≥60 years of age in 2007 and 6.9% in 2008,^2^ similar to a previous study that reported an average prevalence of 7.1% in South America.^3^ Given the expected rise in dementia prevalence, these previous statistics are likely underestimates of the current situation. This epidemiological scenario presents significant challenges for the Peruvian health system, which is characterized by fragmentation, unequal access, frequent staff turnover, and limited specialized and non‐specialized training in dementia care.^4^ Although a new policy was enacted in 2018 as part of a mental health reform (Law No. 30795 “Law to promote prevention, diagnosis, and comprehensive care for Alzheimer's and other dementias”), and in 2025 the National Dementia Plan was published, the implementation of these are still in the initial phases.^5^

**RESEARCH IN CONTEXT**

1. **Systematic review**: It is well recognized that Peru and other low‐ and middle‐income countries have fragmented health systems, and that mapping the different care pathways available to patients in their pursuit of diagnosis and treatment is essential to improving service delivery. In the case of dementia and the Peruvian health system, there is currently no evidence describing the characteristics of these care pathways and patient experiences.
2. **Interpretation**: We present the first mapping of the care pathway through the Peruvian health system for people with dementia, based on patient, carer, and healthcare worker experiences from four diverse sites in Peru. This mapping identifies key points of contact, critical milestones, time intervals between visits, users’ emotional responses, and barriers and facilitators to diagnosis and care.
3. **Future directions**: These findings can strengthen ongoing initiatives to improve dementia care, particularly by highlighting the central role of caregivers in the care of people with dementia, as well as the importance of primary health care. Future studies could apply these methods to understanding the patient journey in other settings, such as other Latin American contexts.

Peru is a highly centralized country. The concentration of specialized services and personnel in the capital city of Lima, combined with system overload, shortage of medicine, consumables, and functioning equipment often forces public and social security beneficiaries to seek private services for diagnostic tests, medications, or specialist care.^6^, ^7^ Among individuals who seek care for a health problem, 18.6% do so at a private pharmacy or drugstore, compared to 14.2% who visit public health facilities and 4.6% who access social security services.^8^ This highlights a fragmented health system with multiple care‐seeking pathways, underscoring the need to understand how people with dementia and their carers experience diagnosis and treatment across different parts of Peru.

This study takes a qualitative approach to exploring the patient journey through the Peruvian health system, drawing on the lived experience of the dyad formed by the patient and their carer, as well as the experiences of healthcare workers who support them. The need for qualitative, experiential evidence is especially urgent in contexts where care for chronic conditions such as dementia is prolonged, emotionally complex, and dependent on multiple actors. Understanding these trajectories not only helps identify barriers but can also highlight opportunities to improve coordination, accessibility, and the overall quality of care.^9^, ^10^, ^11^, ^12^ Although the patient journey approach is aligned with the principles of patient‐centered care, its value in settings like Peru lies in its capacity to expose the tensions between the formal organization of care and the actual experiences of those navigating the health system.^13^ For these reasons, this study aimed to elucidate the patient journey of people with dementia within the Peruvian health system, drawing on patient, carer, and health professional perspectives to identify key moments of interaction with health services, family decision‐making processes, and the institutional, social, and structural factors that shape the care experience.

The study aimed to answer the following research questions:

1. What are the experiences and challenges of providing care and for the unmet needs of people with dementia, their carers, and healthcare workers?
2. What are the most common barriers and facilitators to being diagnosed with dementia from the perspective of people with dementia, their carers, and healthcare workers?
3. What are the most common barriers and facilitators to receiving or providing treatment and management of dementia from the perspective of people with dementia, their carers, and healthcare workers?

## 2 METHODS

### 2.1 Study design

This study is part of the Innovations using Mhealth for People with dementia and Co‐morbidities (IMPACT Salud) study, a 4‐year project with the aim of strengthening health systems in Perú and Latin America using dementia as a tracer condition to inform sustainable, community‐delivered, technology‐enabled innovations.^14^, ^15^ The IMPACT Salud project comprises four distinct work packages; this manuscript focuses on the results of the first work package, specifically a sub‐study about the experiences of people with dementia, outlined in more detail in the work package protocol.^16^ The study adopts a qualitative patient journey methodology to understand the experience of receiving a dementia diagnosis and treatment in Peru for people with dementia (PwD), from the perspectives of PwD themselves, their carers, and healthcare workers. The study is reported in line with the Consolidated Criteria for Reporting Qualitative Studies (COREQ)^17^ (Material S1).

### 2.2 Study setting

The study was conducted in four sites in Peru reflecting the geographical, social, and cultural diversity of the country: Lima, Iquitos, Huancayo, and Tumbes (see Table 1).

**TABLE 1:** Characteristics of the study sites or locations.

Sites	Population size (2024)	Natural region	Illiteracy rate (15+ years old)	Education level (completion of high school; 15+ years old)	Number of CMHCs^a^ in the site
Lima Metropolitan—Lima	1,024,5445	Coast	2%	50.2%	72
Huancayo—Junin	622,063	Highlands (Andes)	5.3%	45.5%	4
Maynas—Loreto	544,777	Amazon Jungle	5.4%	50.1%	4
Tumbes—Tumbes	162,764	Coast	4.1%	48.3%	4

*Source*: Repositorio único Nacional de Información en Salud (REUNIS) + MoH(18).

Abbreviations: CMHCs, community mental health centers.

#### 2.2.1 Peruvian health system context

Peru has three natural regions—the coast, highlands, and jungle—and 55 Indigenous groups, most of whom live in the jungle. Lima, the coastal capital, has a concentration of over 30% of the population.^18^ As a result, many services, such as specialized hospitals and highly trained health professionals, are centralized in the capital. In contrast, the highlands and jungle face major gaps in health infrastructure, qualified personnel, and access to health services.^7^, ^19^, ^20^

Peru's health system provision is divided into public sector subsystems, composed primarily of the Ministry of Health (MoH) and social security department (EsSalud), as well as the private sector.^7^ Each sector has distinct service providers, financing mechanisms, and insurance schemes. The MoH, together with regional governments, administers the country's largest network of primary care facilities. This network uniquely incorporates community mental health centers (CMHCs), a level of service not provided by other subsystems such as EsSalud or private insurance.^6^ The MoH network covers 63.1% of the insured population, followed by EsSalud with 23.4%.^21^

### 2.3 Participant recruitment

The study aimed to recruit 40 participants across the four sites, 10 from each site from three different groups: a representative of the dyad formed by PwD and their carers (either a carer or a person with dementia), and healthcare workers. The inclusion criteria for each participant type are detailed in Table 2. A diagnosis of dementia was required for PwD to participate to ensure that the journey through the health system had reached the point of diagnosis at a minimum. We did not aim to recruit people with mild cognitive impairment (MCI), a syndrome that may progress to dementia over time, because this diagnostic term is rarely employed in the Peruvian health system. Likewise, PwD who scored above 6 on the Pfeffer Functional Activities Questionnaire were not considered eligible to participate as this score indicates a moderate–severe level of cognitive impairment, which could compromise their ability to respond reliably to the study questions.^22^ PwD were also required to report at least one diagnosed comorbidity, as the IMPACT Salud study focuses on these conditions as tracers for strengthening the health system.^14^

**TABLE 2:** Inclusion and exclusion criteria for each participant group.

Participant group	Inclusion criteria	Exclusion criteria	Target per site	Total target
People with dementia (PwD)	Dementia diagnosis: Prospective participants were asked for a self‐reported diagnosis of dementia. Where this was not possible, the carer was asked to confirm this. This diagnosis needed to have been given by a physician in a health facility. Level of functionality: The Pfeffer Functional Activities Questionnaire (PFAQ), Spanish version, was conducted with potential participants (n = 31); participants needed to score below 6 to participate. PwD also needed to have at least one chronic comorbidity, such as hypertension, diabetes, depression, and anxiety, among others.	Unable to communicate with the interviewer due to language barriers. Unable to provide informed consent to participate in this study. Having any disability that impedes communication.	3	12
Carers	People that self‐identified as formal or informal carers of a PwD, including family members that were responsible for taking care of a PwD. Required to have been involved with the process of diagnosis and management of the PwD they care for and be self‐recognized as carers of the PwD for at least 1 year. Carers were eligible to participate regardless of the stage of dementia of the PwD they care for.		3	12
Healthcare workers	Health professionals (family physicians, nurses, and psychologists) and specialists who provide care to PwD at MoH healthcare facilities.		4	16

Abbreviations: MoH, Ministry of Health.

Participants were recruited using a convenience sampling approach,^23^ carried out in two stages. First, healthcare workers who provide care to PwD in primary and secondary MoH healthcare facilities were invited to take part. Once familiar with the project, these healthcare workers acted as points of contact to invite carers and PwD receiving care at the health facilities where they work to participate in the study. This was done either in person or by telephone. Although it was expected that the same number of carers and PwD would be interviewed as representatives of the patient–carer dyad, across all sites there were instances where individuals who had been initially identified as potential participants and expressed interest in taking part in the study were unable to participate because they did not meet the inclusion criteria (see Table 2). The number of these excluded cases was not systematically recorded.

### 2.4 Data collection

Local field teams (groups of two or three field workers) were engaged in each of the four sites. Fieldworkers were based in or originally from the respective areas, which facilitated understanding of the local context. Each field team included one coordinator and at least one additional fieldworker. The coordinator was responsible for handling logistical arrangements and obtaining the necessary administrative permissions from local health authorities to facilitate the research. All fieldworkers were responsible for conducting and transcribing the interviews. In the case of recruiting PwD, the field workers were also responsible for applying the Pfeffer tests. Five of the fieldwork team members were women and four were men. All had prior experience in data collection for health research. Four were psychologists, two were anthropologists, and three were midwives. All fieldworkers received in‐depth training for conducting the study (delivered by the main research team), which included educational sessions on dementia and agism, the Peruvian health system, ethical protocols, and the qualitative data collection methods.

Semi‐structured interview guides with open‐ended questions were developed that focused on all steps a patient goes through to access a typical dementia diagnosis. A specific interview guide was used for each type of participant, taking into account their particular experience (see Supplementary Material of the study protocol^16^). All interview guides explored topics related to understanding the process of seeking a dementia diagnosis and accessing treatment for the condition. The interview focused on three stages of the patient journey, initially termed pre‐diagnosis, diagnosis, and treatment. For each stage, the interviews explored the interactions with healthcare staff, points of contact, experiences and emotions associated with that stage, timing, and barriers and facilitators to accessing care. As part of the inductive analytical process, these initial terms were revisited in light of the empirical findings. The updated terms and their definitions are presented in the results section.

The interviews were conducted in‐person by the fieldwork teams in locations where participants felt most comfortable including their homes, healthcare facilities, and workplaces. The interviews were audio recorded and transcribed with durations lasting an average of 40 min (range 30–100 min). Data collection concluded once the number of participants per site and group had been reached. Given difficulties in recruiting PwD meeting the inclusion criteria across sites, these interviews were supplemented with additional carer interviews. All the interviews except one were conducted in Spanish. One interview in Huancayo was conducted in the indigenous language Quechua (mother tongue of the participant) with the presence of an interpreter. This interview was first transcribed in Quechua and then translated into Spanish prior to analysis.

### 2.5 Data analysis and validation process

Data were analyzed inductively using thematic analysis by the research team, which comprised three social scientists with expertise in qualitative methods and health research and one psychologist trainee. There were three phases to the analysis (see Table 3).

**TABLE 3:** Phases of analysis.

Phase	Main objective	People and teams involved	Inputs	Activities	Outputs	*Additional outputs/impact on participants*
1. Preliminary analysis	To become familiar with the data and develop a preliminary patient journey map to visualize the components of each stage in a graphic format	Research team Communications team for designing the map (using Adobe Illustrator)	Interview audio recordings Interviews, translation and transcripts	Reading and listening to interviews Initial coding and grouping of categories Preparation of summaries for each stage of the patient journey	Preliminary results analysis matrix Preliminary patient journey map	N/A
2. Validation process	To sense‐check and validate the findings with study participants	Research team Sample of participants (*n* = 28) from the four sites (healthcare workers and carers)	Preliminary patient journey map Validation meeting guide	Invitations to validation meetings with participants Virtual and in‐person validation meetings (recorded and transcribed)	Second results analysis matrix Adjustments to the preliminary patient journey map Newly collected data	Spaces for peer‐to‐peer sharing of experiences Carers identified new points of contact, especially during the first two stages Healthcare professionals highlighted new aspects of the pre‐diagnosis stage Carers reflected on barriers and facilitators to accessing care after visualizing the journey
3. Final analysis	To triangulate findings from previous phases to produce the final results	Research team, including a carer representative Communications team for designing the final version of the map (using Adobe Illustrator)	Preliminary results analysis matrix Summaries by stage of the patient journey Validation phase results matrix	Review of both matrices Writing up of findings	Detailed results report Final patient journey map (Figure 1) This manuscript	N/A

In the first phase (preliminary analysis), an initial coding framework was developed based on familiarization with the interview audio and transcripts. Then the research team organized the data into a matrix of preliminary themes using Microsoft Excel (e.g., points of contact, emotions, waiting time). In this stage, the team compared the data across the different types of participants in each theme. This allowed experiences to be compared and contrasted, and similarities or differences to be identified in how participants described the same point of contact with the health system. This phase concluded with the creation of summaries for each stage of the patient journey, which served as the foundation for developing a preliminary patient journey map (Material S2), allowing a visual representation of the identified components of each stage in a graphic format. The map was developed using Adobe Illustrator.

The second phase (validation process) involved presenting this preliminary map to a subsample of 28 participants from across the four sites to sense‐check and validate the preliminary findings and to assess participants’ understanding of them, in order to strengthen the analysis. This phase did not include PwD, as it was not feasible to re‐engage them due to challenges related to availability and health conditions. The validation meetings were conducted by the research team, in group and individual formats, either in person or virtually, depending on participants' availability. Participants were encouraged to reflect on whether the map resonated with their practices and experiences. As a result of this process, an additional matrix was developed to organize the observations gathered and to complement or adjust the findings from the previous phase as necessary. Finally, the third phase (final analysis) first involved integrating the analyses from the previous phases to synthesize the results in a detailed report and update the patient journey map. After this, a final draft of the map was reviewed and amended following consultation with the carer representative in the research team.

### 2.6 Ethics

The study was approved by the Ethics Committee of Universidad Peruana Cayetano Heredia (institutional review board [IRB] number 209080) and the Imperial College London Research Ethics Committee (IRB number 6784708). Authorization from the local health authorities in each study site was also obtained. Written informed consent and/or assent was obtained from each participant before the interviews and after explaining the study details, along with the potential risks and benefits of participation. Participants were also informed that they could withdraw from the study at any time if they wished. In all cases, fieldworkers obtained written informed consent or assent. Specifically in the case of PwD, in addition to their own assent, written informed consent was also requested from one of their carers

## 3 RESULTS

### 3.1 Participant characteristics

A total of 39 participants were interviewed (between 9 and 10 per site): 22 were members of a PwD—carer dyad (either a PwD or a carer) and 17 were healthcare workers. Although the aim was to recruit 12 PwD and 12 carers from separate PwD–carer dyads across sites, due to the difficulties in recruiting PwD, of the 22 participants from PwD–carer dyads the majority were carers (18/22) and only 4 participants were PwD. Interviews were conducted between April and August 2024, and no withdrawals occurred during data collection.

Although the majority of interviewees from the PwD–carer dyads represented in the interviews were carers, in each interview, information was collected about the characteristics of the PwD and the carer being represented. For example, where a carer was interviewed, they were asked information about their relationship to the PwD they cared for, as well as the sex of the PwD and their diagnosed comorbidity/ies (see full list of participant characteristics in Material S3). Across the 22 dyads represented by the carer and PwD participants, caregiving relationships included daughters (13/22), wives (2/22), and sons (2/22) of the PwD. Less‐frequent caregiving relationships included ex‐wives (2/22), grandchildren (2/22), and a paid unrelated carer (1/22). Of the PwD in the dyads, over half were women (12/22), and the majority of carers were also women (19/22). The most frequently reported comorbidity among PwD was hypertension (10/22), followed by depression (5/22) and diabetes (3/22). Five PwD were reported to have two or more comorbidities.

Most healthcare workers interviewed were women (12/17). The majority were doctors (9/17), followed by psychologists (6/17) and nurses (2/17). Ten participants worked at the first level of care (primary care), mainly in CMHCs, whereas seven worked in the second level (secondary care), mainly in regional hospitals.

### 3.2 The three stages of the patient journey

**FIGURE 1:**
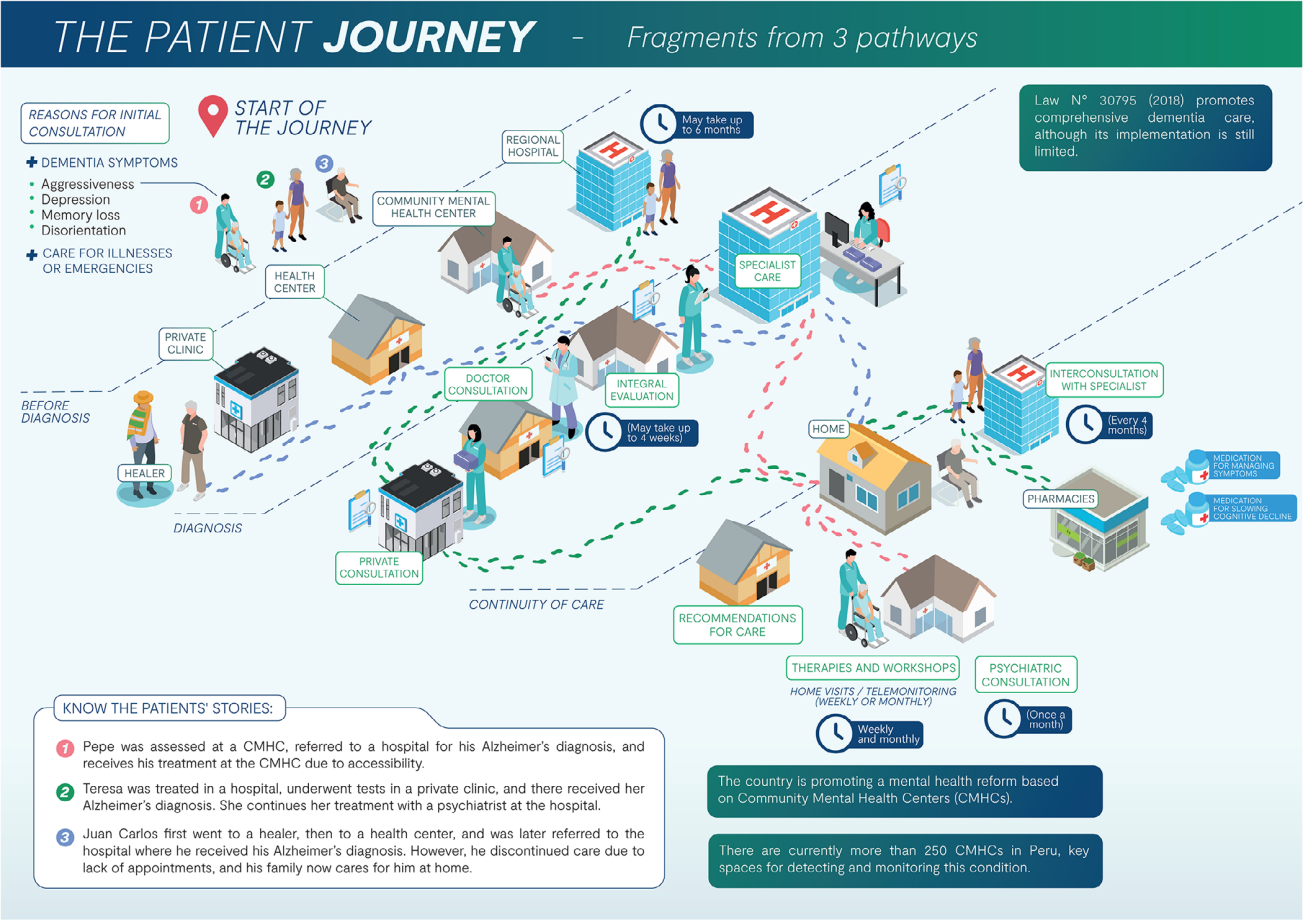
Final map of the patient journey.

The main findings of the study are organized according to the three stages identified in the patient journey, as detailed in the final version of the patient journey map (see Figure 1): before diagnosis, diagnosis, and continuity of care. In each stage, key aspects of the experiences of PwD, carers, and healthcare workers are described in combined summaries. These include the description of access to healthcare, points of contact, the time involved in each stage, the emotions experienced, and the barriers and facilitators encountered.

Each stage was redefined after data collection and analysis, based on the participants’ experiences, as follows. “Before Diagnosis” refers to everything that occurs before the PwD and their family receive a medical diagnosis of any type of dementia. This means that this stage covers the period from when family members begin to notice changes or signs of cognitive decline in the patient, or any event they consider the starting point. “Diagnosis” refers to the moment when a healthcare professional, usually a doctor, communicates the confirmation of a dementia diagnosis to the PwD and/or their family members and carers. This moment may result from the medical opinion of a specialist (such as a psychiatrist, geriatrician or neurologist) or a family doctor. “Continuity of Care” refers to the treatment, of any kind, that the PwD and carers reported receiving. This includes all actions taken after receiving a diagnosis, aimed at managing symptoms, addressing dementia, and/or improving the quality of life of PwD. We chose to use the term “continuity of care” rather than “treatment” to reflect the diverse and often parallel care pathways experienced by PwD and their carers, across different locations and levels of the Peruvian health sub‐systems.

#### 3.2.1 Before diagnosis

Participants described how help‐seeking for dementia care often begins in response to a household crisis, such as a fall due to disorientation or violent behavior toward relatives. Repeated incidents, such as not recognizing family members, perceiving theft or verbal abuse, leaving stoves on, or doors open, create household stress and caregiving strain. These events typically trigger medical consultation, which was often circumstantial and shaped by the family's knowledge of their local health system:

“When I saw his jealousy getting worse—he kept asking me why I was taking so long or if I was with someone else, things like that—that's when I thought, ‘this is really bad, he's lost it’, I think I even said, ‘no, *no… I'm taking him to the hospital’”*. Carer from Iquitos (wife)

Participants described that during this stage, consultations may take place with general doctors, health specialists like endocrinologists, or at health facilities, for example, when PwD are attending appointments for the management of other symptoms and illnesses. Table 4 details the key elements identified at each level of the health system for this stage.

**TABLE 4:** Key aspects of the patient journey related to the “before diagnosis” stage.

Location where first contact with the health system occurred	CMHC (Primary care)	Health centers (Primary care)	Hospital (Secondary care)	Specialized hospital (Tertiary care)—only in Lima
Healthcare workers involved	Nurse or nursing assistant; Psychologist; General practitioner	Nursing assistant; Nurse; General practitioner	General doctor; Specialist	Specialist
Time between symptom onset and first healthcare contact (range)	1–2 years	5 months to 2 years	1–5 years	1–10 years
Tools or exams	Intake process: consists of the first contact, during which the mental health nurse guides and informs the user about the procedures and requests their informed consent for care. Cognitive impairment screening tests	Cognitive impairment screening tests	Cognitive impairment screening tests	Cognitive impairment screening tests; Diagnostic support through psychological tools, blood tests, and neuroimaging and CT scan or MRI
Perceptions and emotions of PwD	Irritability during the evaluation. Denial due to not perceiving it as an illness. Confusion caused by incomplete or contradictory information about symptom understanding. Exhaustion due to hospital administrative procedures. Sadness caused by the patient's symptoms.			
Perceptions and emotions of carers	Concern about management of comorbidities and the overall health of the PwD. Stress related to caregiving and household finances.			
Barriers from the health system	Not all CMHCs have psychiatrists. No neuroimaging and CT scan or MRI examinations available.	Lack of adequately trained personnel to guide individuals in the diagnostic process.	Lack of adequately trained personnel to guide individuals in the diagnostic process. Long wait times to get an appointment. Health personnel not trained to care for PWD. Difficulty of access for people living in rural areas.	Long wait times to get an appointment. Services are mainly available in the capital city, Lima.
Barriers related to family circumstances	Carers’ limited time, financial constraints, and physical limitations			
Facilitators	Short wait time to get appointments. Kind and respectful treatment by healthcare staff Free service covered by public health insurance “Seguro Integral de Salud” (SIS)	Short waiting time for the first. appointment Proximity to people's homes Free service covered by SIS	Free service covered by SIS	Trust in the information provided by the specialist Free service covered by SIS

Abbreviations: CT, computed tomography; MRI, magnetic resonance imaging; CMHCs, community mental health centers.

##### First points of contact

The data showed that PwD and their carers have varied first engagements with the health system, differing with regard to the levels of care accessed, types of consultation, health personnel involved, and other aspects. Care may begin at the first or second level of care, with the most frequent first point of contact being Primary Care Centers (including CMHCs). PwD access hospital care through paid outpatient consultations, emergency visits to public hospitals or private clinics, or referrals from specialists who are managing other diagnosed comorbidities. Hospitals were the second most frequent initial contact. Hospital care is often sought, both in and outside the capital, due to perceptions that primary care is inadequate for addressing dementia symptoms, or following crisis events such as falls or accidents, that may have been perceived as linked to cognitive or behavioral alteration and requiring urgent medical attention.

With regard to accessing primary care facilities, the data suggest that PwD generally attend these facilities to receive treatment for comorbid conditions (e.g., hypertension or diabetes), and are then advised by their physician to visit a CMHC after signs of cognitive impairment are observed. It was also noted that some PwD directly access CMHCs based on exposure to radio announcements or personal recommendations regarding mental health services.

The time from the first signs of cognitive decline to seeking healthcare was reported to range from 1 to 10 years, with some variation across sites. In all sites, lack of awareness and normalization of cognitive or behavioral symptoms as simply part of the aging process was identified as a major cause of diagnostic delays. In cases where care is sought sooner (for example within 5 months of onset), this was explained to be driven by family history of dementia or prior disease awareness.

According to the participants, key actors in the pre‐diagnosis phase include family members and carers, who first notice signs of cognitive decline and encourage medical consultation. Primary care professionals and hospital staff also play critical roles in recognizing signs of cognitive decline and facilitating formal diagnoses, often in the course of routine appointments for other conditions. As noted in the following quote, participants from Huancayo and Tumbes described that alternative medicine and traditional healers are initially consulted, particularly when symptoms are attributed to cultural or supernatural causes.

“Because of the illness, I came [to the health center]—my daughter brought me, dear. I was feeling very unwell, dear. Even when the traditional healer treated me, I didn't get better. (…) [Later] I went to the health center with my daughter, with my neighbour, dear.” PwD from Huancayo

##### Emotions reported at the beginning of the journey

Participants talked about how carers and families experience worry, sadness, and stress due to noticeable changes in the PwD. Many initially normalize signs of dementia as part of aging, but concern grows as these worsen. For those navigating hospitals, additional emotions such as irritability, confusion, and exhaustion were reported, often associated with long waiting times and negative consultation experiences:

“The other day, the technician called me and said, ‘Come to the center so you can book your appointment.’ I went, happy, thinking, ‘Now I'll finally get my appointment, they'll give me my treatment.’ The day I went, I got to the counter, and they told me, ‘Go to window three.’ I went there, waited in a huge queue, and when I finally got to the window, they said, ‘No, ma'am, you'll get a call on your phone’. And that's a lie—last year it was the same, and they never called me. I started getting upset, and I yelled at them, asked why they don't attend to me just because I'm a senior. I told them my back hurts, I fell. But even after all that, they didn't see me. I went to see the doctor, but she wasn't there—just another technician.” PwD from Lima

##### Barriers and facilitators

Barriers to seeking care revealed in the findings include widespread lack of dementia knowledge among PwD, families, and some healthcare professionals, particularly at the primary and secondary care levels. Misconceptions about dementia as an untreatable, natural part of the aging process were reported to delay care‐seeking. Healthcare professionals’ lack of knowledge often leads to failure to refer patients to specialists or discouragement of specialized care.

Additional barriers included carers’ limited time, financial constraints, and physical limitations. Families often compensate for these gaps by experimenting with alternative routes—like relying on informal advice—illustrating early forms of agency that shape the rest of the journey. A key facilitator is the country's public health insurance “Seguro Integral de Salud” (SIS), which provides free access to care across all levels, particularly at CMHCs.

#### 3.2.2 Diagnosis

Interviewees indicated that a diagnosis of dementia usually occurs at the CMHC (primary care) or in hospitals (secondary care). A few diagnoses were also received at specialized hospitals or in private clinics (outside the public health system). These points of contact varied in terms of the professionals involved and the tools used for diagnosis, as well as in the waiting times, financial cost, perceptions and emotions about the treatment and quality of care. Table 5 shows the elements identified at each place where diagnoses are received.

**TABLE 5:** Key aspects of the patient journey related to the ‘diagnosis’ stage.

Place within the health system where diagnoses are obtained (Point of contact)	CMHC (Primary level of care)	Hospital (Secondary level of care)	Specialized hospital (Tertiary level of care)—Lima	Private clinics (Outside the public health system)
Healthcare workers involved (Point of contact)	Multidisciplinary team: Psychiatrist or family doctor, Psychologist, nurse	Psychiatrist; Neurologist; Geriatrician		
Wait time for diagnosis appointment	1–2 weeks	1–2 months (up to 6 months in Lima)	1–6 months	1 day to less than a week
Tools or tests (Point of contact)	Mini‐Mental State Examination or another brief cognitive screening test; Referrals are made to other levels of care for complementary examinations	Brief cognitive screening test (MMSE, Clock drawing test or MoCA); Laboratory tests (e.g., vitamin B12, thyroid function, infection screen); neuroimaging and CT scan or MRI		The same diagnostic tests as those used at MoH secondary and tertiary levels are available
Perceptions and emotions of PwD	Not clearly reported	Discomfort, boredom, exhaustion, irritability		Not clearly reported
Perceptions and emotions of carers	Shock, anxiety, sadness, worry, relief, reorganization of routines, economic stress, trust, security	Same as CMHCs, except for “trust” and “security”		More efficient in obtaining a diagnosis
Barriers to obtaining a diagnosis related to the health system or family circumstances	No barriers reported	Bureaucratic and slow processes; shortage of professionals, especially outside Lima; part‐time work arrangements; lack of supplies for tests; negative or disrespectful treatment of older adults		Financial cost
Facilitators to obtaining a diagnosis related to the health system or family circumstances	Comprehensive care described as “good” and “complete”; kind and understanding staff; family financial support	Family financial support (transportation or tests)		Fast attention, family financial support

Abbreviations: CT, computed tomography MRI; magnetic resonance imaging; CMHCs, community mental health centers.

##### Pathways and places of diagnosis

When a diagnosis is received at the CMHC, patients undergo a comprehensive evaluation that includes the participation of healthcare workers from Nursing, Psychology, and Psychiatry or Family Medicine departments (depending on availability). Scheduling an appointment at the CMHC was described as quick: both health personnel and patients and carers reported waiting times of between 1 and 2 weeks across sites. Healthcare workers noted that sometimes referrals are made to hospitals for complementary tests (e.g., computed tomography (CT) scans, blood tests, etc.).

“The first appointment was with the psychologist, who talked to us; they also did a test and told us that yes, she [the PwD] has dementia. Then they said, ‘Now we'll refer you to psychiatry so they can prescribe the medication.’ It was about a week later that we were seen.” Carer from Huancayo (daughter)

In hospitals, diagnoses of dementia are usually made by a specialist (Psychiatrist, Neurologist or Geriatrician), generally during the second consultation. All participant groups indicated that in these cases, complementary tests were requested by the doctors, such as laboratory tests, CT scans, and especially in Lima, magnetic resonance imaging (MRI). The wait times for an appointment at the secondary or tertiary level, which is accessed via referral, were found to vary by site. In Huancayo, Iquitos, and Tumbes, the average wait times for referrals were reported to be 1 to 2 months, whereas in Lima they extended from 3 to 6 months. However, even when referrals are issued, the data revealed that most patients do not end up receiving the complementary tests. Healthcare workers explained that hospitals do not have the supplies needed for these tests, so patients are encouraged to do them privately in order to avoid delaying the diagnosis. As one healthcare worker explained, hospitals frequently face shortages that slow down the diagnostic process. These tests usually include neuroimaging exams (CT or MRI) or psychological tests to assess cognitive decline. The data suggest that those who could afford it reported attending private laboratories to obtain the tests and then returning to the public facility with the results. Consequently, where the PwD and their family do not have the economic resources to afford such private tests (the majority of cases), the PwD receives a diagnosis that is based on clinical judgment alone.

“We only have CT scans, which help us a lot at first to see if there's hippocampal or frontal atrophy, but ideally, we should have an MRI. Unfortunately, *as a tertiary hospital*, *we don't have a magnetic resonance scanner (…) these processes should be faster. People shouldn't have to wait two months for an image or go out looking for someone who can do lab tests because the hospital doesn't have the reagents.”* Healthcare worker from Lima

As above, a small group reported that they received their diagnosis in a private clinic or consulting room. These cases happen as “leakages” from the public health system: patients, while waiting to be seen at the secondary level, seek private care to speed up the start of treatment. They described that this was possible thanks to financial support they receive from other family members.

An interesting finding is that diagnoses do not necessarily happen only once. There were cases discussed of parallel or repeated diagnoses. This was mainly related to two factors: first, the fragmentation of the health system, which leads people to consult with more than one institution; and second, the desire to seek confirmation or reassurance about the diagnosis, especially when families had limited trust in the first assessment. For example, patients who received a diagnosis at an EsSalud hospital later went to a CMHC (at MoH) and were diagnosed again. Others, after being diagnosed at a private clinic, attended their public hospital appointment and received a second diagnosis. There were also cases where family members did not accept the initial diagnosis, so they sought a second opinion at a specialized hospital or private clinic:

“Oh yeah, what happened was that at the health center [Name of the center], in order to get another appointment at the hospital—since that's where the specialists are—we needed a referral from the doctor. He gave us the referral, but something was wrong with it—either the diagnosis, or maybe his signature or stamp, I'm not sure. So, at the hospital they wouldn't give us the appointment because of the referral, and they told us we had to go back to the health center. But when we went back, the doctor wasn't on duty anymore, and then over the next few weeks my mum got worse, so we ended up just leaving it and taking her to a private clinic instead.” Carer from Huancayo (daughter)

##### Emotions and reactions to the diagnosis

Carers reported emotions such as shock, anxiety, sadness, and worry. Health professionals also recognized that, along with these feelings, some carers expressed relief at finally knowing what was happening with the person they were caring for. Following the diagnosis, many families began to think about how they might reorganize daily routines, time, and financial resources in response to anticipated care need, which was described as stressful.

The PwD reported that at this stage there were feelings of discomfort, boredom and fatigue associated with wait times and their interaction with specialists at hospitals within secondary and tertiary care. In addition, healthcare workers noted that some patients reacted negatively upon receiving their diagnosis, showing signs of aggression. In contrast, due to the attitude and information provided by staff at the CMHC, carers and family members reported feeling trust and safety when receiving the diagnosis at these facilities. As one healthcare worker explained, this is linked to the center's design and the familiarity of the approach to care:

“What patients are looking for is speed, immediacy, and, in addition to that, there is also the issue of the professional themselves—they feel that here, *in the community mental health centers*, they are treated better; the care is more humane, more direct, *more familiar [than the Hospital]*, and the very environment is also designed for that.” Healthcare worker from Lima

##### Barriers and facilitators to receiving a diagnosis

The main barriers to diagnosis identified included barriers to obtaining appointments linked to bureaucratic and slow processes in MoH services and health facilities. In sites such as Huancayo, Iquitos, and Tumbes, this issue is worsened by the shortage of specialists—such as psychiatrists, geriatricians, and neurologists—in regional hospitals and by the fact that some work only 15 days per month, leaving the other half of the month without services in these specialties and forcing the family to look for a private consultation, even with the same provider from the public system:

“So the doctor says to me (…) ‘You know what, madam? The gentleman has senile dementia,’ she says, ‘and what I'd actually recommend is… (…) that you go to the psychiatrist,’ she says, ‘so they can give you some pills or some medicine that might hold it back a bit,’ she says, ‘so he can relax, feel a bit better (…).’ And she tells me, ‘The psychiatrist here at the hospital doesn't work every day, only once a week, but if you want to see him, he's at the [name of Private Clinic] every day,’ she says. So I said, well, I wanted to know quickly what medicine he could give or what he was going to tell me, right? So I just went—we went to the [Private clinic]”. Carer from Iquitos (daughter)

In Iquitos and Lima, some carers reported negative experiences with specialists in hospitals, describing a lack of patience toward older adults and mistreatment, including physical restraint measures, especially when symptoms of cognitive impairment made it difficult for the PwD to understand the tests or procedures being conducted. These experiences were described as leading to discomfort and distrust, prompting some caregivers to discontinue care at these facilities or return to primary care facilities for further needs.

*“The care at the hospital was terrible*—*the way they treat patients just isn't right. And especially for us family members*, they don't let us be near the patient while they're admitted. *In* my case, there was serious neglect. My mother [PwD] was admitted just for a stroke, but because they didn't take proper care of her, she ended up with pressure sores on her back. She would scream, *and what they did was tell her to shut up and tie her up. They tied her down. I found out through someone I know who also goes to that hospital*—*they told me what was happening*, and I went right away. When I got there, I found my mother tied up in a straitjacket.” Carer from Lima (son)

Another recurring barrier was a lack of supplies to carry out the complementary tests requested by specialists (reagents, CT scanners, etc.). This was also a factor in families feeling forced to go to private laboratories, incurring higher costs, or, in some cases, deciding not to undergo the tests. These difficulties were reported to cause emotional, physical, and economic exhaustion for both PwD and carers.

The main facilitator was the care provided at the CMHC, which was perceived positively. PwD and carers described it as “good,” “comprehensive,” and “complete.” Psychology staff were highly appreciated, being described by PwD and carers as “kind” and “understanding,” and consideration was given to the specific needs of users, including being seen before other non‐older adult individuals. This approach greatly influenced participants’ preference for being treated at CMHC rather than hospitals:

“[I received good care], I went first, right? I told them everything, and then, since I'd already told the nurse I'd bring her [PwD] on a specific day, they were already expecting us, and she was seen straight away (…) and since then, yes, the psychologist sees her every two weeks, and the other specialist, and the psychiatrist sees her once a month—every month.” Carer from Tumbes (daughter)

Finally, an important facilitator was family support, particularly in covering expenses related to diagnosis, such as complementary tests or transportation, which in some cases allowed PwD to receive diagnosis from private clinics, and in many cases for families to sustain the care process.

#### 3.2.3 Continuity of care

The data revealed that, after receipt of a dementia diagnosis, care is provided by carers at home, with their most frequent interaction with the health system occurring through CMHCs, followed by general hospitals and specialized hospitals, and complemented via private pharmacies. These do not always coincide with the places where the first diagnosis was received. One factor in determining where care is accessed was the instructions provided by the specialist who referred the PwD, for example, from a hospital to a CMHC. In some cases it was a decision of the PwD and their family, who, after obtaining a diagnosis and treatment prescription from a hospital, may look for health centers located in more convenient areas, which may be in primary or secondary care.

The data suggested that some PwD, after receiving a diagnosis, only go to private pharmacies to purchase medication until their prescription expires. Following expiry of a prescription, PwD and their carers reported buying medications that do not require a prescription. Table 6 presents the key elements identified in the continuity of care stage.

**TABLE 6:** Key aspects of the patient journey related to the “continuity of care” stage.

Point of contact	CMHCs (Primary care)	Hospitals (Secondary level of attention)	Specialized hospitals (Tertiary care, Lima only)	Private outpatient clinics (Outside public system)
Healthcare workers involved	Psychologist; Nurse; Psychiatrist	Psychiatrist; Neurologist; Geriatrician	Psychiatrist; Neurologist; Geriatrician	No cases were identified of PwD receiving treatment in private clinics
Frequency (Treatment starts once PwD obtains prescribed medication, typically at diagnosis)	Follow‐up visits occur weekly—biweekly			
PwD perceptions and emotions	Group sessions provide enjoyment and engagement for PwD. Long wait times on the same day cause fatigue	Long wait times on the same day cause fatigue.	Long wait times on the same day cause fatigue.	Not clearly reported
Carers’ perceptions and emotions	Financial strain to afford medication. Carer stress from scheduling conflicts. Carer relief from observed medication benefits			
Barriers from the health system	No availability of dementia‐specific medications. Limited availability of drugs for dementia symptoms. Workshops not tailored to dementia. Attendance dependent on carer availability.	No availability of dementia‐specific medications. Limited availability of drugs for dementia symptoms. Attendance dependent on carer availability. Limited access in rural areas.	No availability of dementia‐specific medications. Limited availability of drugs for dementia symptoms. Attendance dependent on carer availability. Tertiary care limited to Lima.	Expensive medication and treatment
Barriers related to family circumstances	Carers’ limited time, financial constraints and complicated transportation to health care facilities			
Facilitators	Good geographic access. Home visits available. Positive provider attitude. Free care under SIS.	Free care under SIS.	Free care under SIS. Trust in specialized providers.	Trust in specialized providers. Rapid access to labs/neuroimaging for treatment monitoring.

Abbreviations: SIS, comprehensive health insurance/seguro integral de salud; CMHCs, community mental health centers.

The treatment follow‐up was reported to be typically led by a specialist such as a neurologist, psychiatrist, or geriatrician, sometimes involving other professionals in cases of comorbidities. This was found to depend on which specialist made the diagnosis. We did not find any cases or opinions describing a change of specialist within the same hospital. However, we did find cases where the patient attended psychology services for testing (as diagnostic support) and then returned to the specialist physician.

On the other hand, we also found examples where treatment proceeded at the primary care level. CMHC's were found to offer a standardized care package for the PwD, that includes monthly psychiatry consultations, psychological therapy, occupational therapy, and workshops adapted to the patient's needs, with limited possibilities of home visits and telemonitoring.

##### Types of treatment at healthcare facilities

Clinical guidelines for the treatment of dementia were not implemented in Peru at the time of the interviews. The data suggest that most patients (17/22) were receiving pharmacological treatment focused mainly on controlling symptoms such as anxiety, aggressiveness, and mood disorders. The most common medications discussed include anxiolytics and sedatives (the most frequent was clonazepam), and antipsychotics (the most frequent were haloperidol and quetiapine). Only six patients received specific medications for cognitive decline, mainly memantine. The lack of availability of medicines in public facilities was reported to force some patients to purchase them in private pharmacies:

“(…) that's what the psychiatrist told me—he said that the health center doesn't have that pill [referring to a medication for cognitive decline], and well, (…) we have to buy it, right? That's what the psychiatrist told me: we have to buy it at the pharmacy, right?” Carer from Iquitos (daughter)

Non‐pharmacological treatment was reported in 12 PwD of the dyads represented in the sample and consisted mainly of psychological therapy, which most participants received, as well occupational therapy, and general recommendations for daily management at home, such as maintaining physical and mental activity, a healthy diet, and structured routines. Psychological and occupational therapy were reported to take place at the CMHC and in some cases at hospitals, as both individual and group sessions. Its duration was ≈7 weeks, and adherence, according to interviewees, depended on the availability of time and resources of the carer.

The waiting times to get an appointment with specialists in hospitals can be very long, from 3 to 6 months, reportedly causing abandonment of follow‐up or preference for the CMHC, which offer appointments with greater speed and proximity. In some cases, patients can take up to a year to resume care after missing scheduled appointments. Common reasons for abandoning or pausing treatment, regardless of the level of care, included difficulties in mobility, economic expenses related to transport and medicines, lack of time on the part of carers, avoiding reprimands from doctors due to discontinuity in medication intake, as well as the perception of clinical stability of the patient. In certain sites such as Iquitos, the lack of adaptation of psychological therapies to patients with visual disabilities was specifically mentioned.

It was highlighted by doctors that decisions on frequency of appointments are usually taken at their discretion according to the stage of the disease. Often, when the diagnosis has been received late and cognitive decline is advanced, the next appointment is scheduled for 2 to 4 months later, based on the physician's perception that there is little that can be done to help the patient. These time intervals are perceived by patients as reasons for having discontinued their follow‐up visits at the hospital, particularly when they do not live in the same city as the hospital:

“What we [healthcare workers] usually do is tell the patient to come back every three months, because that's how long the medication lasts in some cases. So, they're supposed to come in for a check‐up every three months, but only a few actually follow through with that. (…) Sometimes it's because they don't have family support—their relatives are working, they don't have anyone, or they live alone. There's also abandonment, and sometimes it's just hard to get an appointment with the services.” Healthcare worker from Huancayo

##### Emotions and reactions related with the treatment and care

The data suggest that no psychological or other support programs have been systematically implemented for carers of people with dementia. What we found are isolated and discretionary efforts by psychologists in some CMHCs, who invite carers to attend therapy sessions addressing carer burden.

Carers reported experiencing a range of emotions in this stage. The emotional and physical burden of reorganizing daily routines and responsibilities was particularly emphasized, with frequent expressions of stress, fatigue, and financial concerns related to medical expenses. However, they also reported feelings of calmness and satisfaction, especially within the household, when observing improvements in the patient's symptoms such as improved management of anger or sleep. In some interviews, this perceived improvement was cited as a reason for not pursuing further diagnostic procedures such as neuroimaging, or for discontinuing or not purchasing medications aimed at treating cognitive decline.

Regarding experiences of care, PwD mainly expressed positive emotions about the treatment received at CMHCs, which was characterized as empathetic and humane. Nevertheless, in their experience of receiving treatment in hospitals, they reported feelings of frustration and irritability due to long wait times to enter the consulting room on the day of their appointment.

##### Barriers and facilitators for the continuity of care

Multiple barriers to effective continuity of care were identified in the data. As in the case of the diagnosis pathway, the lack of medicines for cognitive decline in public facilities was frequently mentioned as a major constraint, often compelling patients and carers to make costly purchases from private pharmacies. In addition, the scarcity of specialists, long wait times for appointments, and poor treatment in some centers discouraged treatment adherence (see subsequent quote). Other difficulties include complicated transportation to health care facilities, associated costs, and lack of training and empathy of healthcare staff toward patients and carers. The resources available to PwD and their families also constitute a barrier, with limited financial means and carers’ time constraints being the most common.

*“We haven't been able to go to more appointments because we missed one [after the diagnosis, due to time and work], and now we have to get a new one. But to get it, you have to line up really early*—*basically stay up all night at the hospital*—*and I just can't do that with my son. I also can't send my 80‐year‐old dad to wait in line either. I've sent him before for his back, but he ended up getting worse, and now I have to take care of both of them. It's… honestly, it's just too hard for me.”* Carer from Huancayo (daughter)

Interviewees highlighted factors that support continuity of treatment, especially the positively rated treatment received at CMHCs, ease of obtaining regular appointments, and, in some cases, the possibility for carers to collect medicines directly when the patient cannot attend. The active involvement of family members in monitoring and reporting symptoms also contributes significantly to the proper management of the disease, facilitating adherence and continuity of treatment.

These patterns reveal that the continuity of dementia care in Peru depends largely on the initiative of families and their ability to coordinate across fragmented health services, compensating for institutional gaps through informal problem‐solving.

## 4 DISCUSSION

This study explored the patient journey of PwD in Peru, identifying key moments including first contact with the health system, the diagnosis process, and treatment delivery. The first study of its kind in Peru, this study provides a person‐centered approach to understand the dementia patient journey, highlighting family decision‐making processes and the institutional, social, and structural factors that shape the dementia care experience.

We observed a duration ranging from 1 to 10 years between initial onset of symptoms and accessing support at a health facility, and an additional 6 months for obtaining a diagnosis, often resulting in diagnoses made at advanced stages of dementia. Long wait times hinder access to healthcare services, forcing users to navigate multiple points of care and placing strain on the health system.^9^, ^10^, ^11^ This is consistent with findings from LMIC studies of non‐communicable disease, which identify poor coordination across levels of care and the absence of defined care pathways as systemic barriers to achieving effective care continuity.^24^

The diagnosis stage was highlighted as a critical point in the patient journey for PwD that can either facilitate or hinder their integration into the healthcare system. Treatment continuity is influenced by the accessibility of the health facility providing follow‐up care, the availability of pharmacological treatments, and wait times for subsequent appointments. Similarly, a Peruvian study on the patient journey following stroke associated the lack of structured follow‐up with treatment discontinuity.^25^

From the outset of the journey toward dementia diagnosis and treatment, patients and their carers bear responsibility for navigating a fragmented health system and accessing care aligned with their available resources. In some cases, this pathway was pursued autonomously, beyond clinicians’ recommendations. This often requires families of PwD to combine multiple strategies, including home‐based care, adjusting schedules, sharing costs among relatives, and changing employment or health insurance arrangements. Previous research documents similar forms of adaptiveness, including “connecting touchpoints”^26^ within the Canadian health system. In the United States, the perseverance of carers in seeking a reliable diagnosis has been emphasized.^27^ In contrast, researchers in China observed skepticism toward dementia diagnoses among younger individuals, contributing to ongoing navigation within the health system.^28^ Our findings align with research on the costs of elder care in LMICs, highlighting Peruvian, Mexican, Chinese, and Nigerian carers’ engagement in “bricolage,” combining formal and informal strategies to manage care‐related expenses.^29^

These findings represent a theoretical contribution that conceptualizes patient journeys as non‐linear, iterative, and adaptive.^25^, ^27^, ^28^, ^29^ In Peru, the journey of PwD is shaped by navigation across multiple systems, levels of care, and formal and informal strategies in response to changing needs, uncertainties, and system barriers. This “bricolage” reflects health system fragmentation and the role of carers’ strategies in sustaining trajectories in the absence of clearly defined pathways. Recognizing this journey is crucial for providing a realistic understanding of how health systems are experienced in LMICs.

Across the patient journey, emotions of carers function less as isolated reactions and more as forces that organize actions and decisions. Prior to diagnosis, uncertainty and denial are intertwined with worry and frustration, as families move between attributing symptoms to “normal aging” and mounting distress when behavioral changes disrupt everyday life. These frequent attributions of “normal aging” can be understood through the lens of agism.^30^ Our findings align with agism research, identifying sociocultural patterns within families that delay dementia care‐seeking.^31^, ^32^, ^33^, ^34^, ^35^, ^36^ The impact on professionals who provide care to older adults has also been recognized,^37^, ^38^, ^39^ suggesting that agist attitudes among healthcare professionals can contribute to the normalization of cognitive decline, negatively shaping clinical judgment and attitudes toward older adults. Although our study did not find evidence of agist attitudes among healthcare professionals, if these exist in the Peruvian healthcare system they would likely exacerbate observed issues surrounding lack of knowledge about dementia and treatment protocols as well as other health system constraints.

The diagnostic stage carries the greatest emotional intensity: carers experience shock, sadness, and anxiety, alongside relief at finally naming what had become unmanageable. This relief is fragile and dependent on interactions with professionals. Following diagnosis, emotions shift into a chronic strain, marked by exhaustion, financial pressure, and social isolation. These findings align with studies showing how the pursuit of a more accurate diagnosis of dementia and other non‐communicable diseases, and access to pharmacological treatment, increases carer stress^9^, ^24^, ^28^ These emotions underscore how caregiving for dementia in Peru is sustained less by institutional support than by the family's emotional labor and their persistence and resilience. Similar to research linking caregiving to improved carer–patient relationships,^27^, ^40^ we observed cases where carer stress decreased following improvements in symptoms from medical treatment that led to more stable home environments.

### 4.1 Policy implications

The Peruvian government published its National Dementia Plan in November 2025,^41^ an important step toward the implementation of the “Alzheimer's and other dementias law” enacted in 2018.^5^ The Plan addresses a context in which care depends on individual initiative and family resources rather than institutionalized pathways for diagnosis, treatment, and long‐term support. It establishes budgets, targets, and lines of action, including training for healthcare personnel and support for carers. However, it does not address medication availability for cognitive impairment in public pharmacies, or limited access to MRI for dementia diagnosis, identified as critical health system gaps.

Moreover, the study underscores the potential of CMHCs as existing entry points for dementia care within the public system. Their multidisciplinary model, emphasis on psychosocial support, and positive reception among carers suggest that they could serve as the structural backbone for decentralized dementia services. The Chilean experience in designing and implementing its National Dementia Plan, which relies on primary care, is a relevant example.^42^ Policymakers should prioritize strengthening CMHC capacity, expanding coverage, integrating dementia‐specific guidelines, and linking them to other services through formal referral and counter‐referral mechanisms.

However, strengthening primary care alone is insufficient. Dementia is a neurological condition caused by brain disease, often associated with physical symptoms that may be overlooked in mental health settings. As scientific knowledge advances, diagnosis and management are likely to become increasingly neurological in nature. Moreover, dementia predominantly affects older adults and is commonly accompanied by multiple co‐morbidities, whose diagnosis and management are substantially shaped by the presence of dementia. Therefore, dementia care requires a highly integrated approach, which can be complicated without systematically involving primary and secondary care, as well as specialized hospitals.

### 4.2 Strengths and limitations

A key strength was the development of a patient journey map for PwD within the Peruvian health system. This served as a visual tool that synthesized the findings into a coherent graphical representation. The study included multiple perspectives from different sites on the entire care pathway, providing a diverse view of this trajectory. Local field staff, whose contextual knowledge enriched the understanding of site‐specific dynamics and facilitated the analytical process, carried out data collection. Our partnership with the MoH facilitated access to officials and healthcare personnel across all levels of care. Finally, the implementation of a validation process proved valuable for providing participants with a space to share, discuss, and reflect on their lived experiences, often for the first time.

This study has several limitations. The number of interviews conducted varied across sites, reflecting some challenges in recruiting PwD who met the inclusion criteria. Fieldworkers reported that several PwD who expressed interest in participating either did not meet the inclusion criteria, because they scored above 6 on the Pfeffer test, or lacked a carer able to provide consent, reflecting situations of abandonment. As such, the perspectives of PwD are underrepresented, with interviews conducted primarily with carers due to communication or cognitive difficulties of the PwD.

This study is limited to formally diagnosed PwD in MoH facilities. Consequently, it excludes rural populations, individuals utilizing other systems such as EsSalud, and those with informal or no diagnosis. Another limitation was recall bias among carers and PwD. On average, diagnostic experiences had occurred 3 years earlier and were shaped by subsequent perceptions and difficulties in recalling details. Most participants remembered the behavior and attitude of the physician, whereas recollections of initial symptom onset were vague due to the time that had passed.

## 5 CONCLUSIONS

This study suggests that there is not a singular, coherent care pathway for dementia in the Peruvian health system. Instead, PwD and their families navigate a variety of routes, encountering diverse and often fragmented points of contact. This reflects a lack of coordination across levels of care. Despite this, families demonstrate agency and adaptability, often making strategic decisions about referrals, counter‐referrals, follow‐up appointments, and medication access, within and beyond the public system. However, this autonomy is shaped by the resources available to the family and therefore impacts not only the search for an adequate diagnosis but also adherence to treatment.

Finally, findings suggest that CMHCs are the only health facilities that are structurally, procedurally, and professionally prepared to care for PwD across the four sites. However, in the absence of a formalized and public entry point to the health system for dementia care, these centers are not widely recognized as a first point of contact.

## AUTHOR CONTRIBUTIONS

**Conceptualization**: María Lazo‐Porras, Jemma Hawkins, Francisco Jose Tateishi‐Serruto, Miriam Lúcar‐Flores, Christopher Butler, María Sofía Cuba‐Fuentes, J Jaime Miranda, Antonio Bernabe‐Ortiz, Francisco Diez‐Canseco, Maria Kathia Cardenas, Graham Moore, Filipa Landeiro, Jose Carlos Vera Tudela, Lee White, Rafael A Calvo, William Whiteley. **Data curation**: Daniela Rossini‐Vilchez, Francisco Jose Tateishi‐Serruto, Silvana Perez‐Leon. **Analysis**: Daniela Rossini‐Vilchez, Francisco Jose Tateishi‐Serruto, Silvana Perez‐Leon, María Lazo‐Porras, Jemma Hawkins, Guillermo Almeida Huanca, L. Carolina Olaya Silva. **Funding acquisition**: María Lazo‐Porras, Jemma Hawkins, Christopher Butler, María Sofía Cuba‐Fuentes, J Jaime Miranda, Antonio Bernabe‐Ortiz, Francisco Diez‐Canseco, Maria Kathia Cardenas, Graham Moore, Filipa Landeiro, Jose Carlos Vera Tudela, Rafael A Calvo, William Whiteley. **Investigation**: Daniela Rossini‐Vilchez, Francisco Jose Tateishi‐Serruto, Silvana Perez‐Leon, Jemma Hawkins, María Lazo‐Porras. **Methodology**: Jemma Hawkins, María Lazo‐Porras, Francisco Jose Tateishi‐Serruto, Silvana Perez‐Leon, Miriam Lúcar‐Flores, Jose Carlos Vera Tudela, Maria Kathia Cardenas. **Supervision**: María Lazo‐Porras, Jemma Hawkins. **Validation**: Daniela Rossini‐Vilchez, Francisco Jose Tateishi‐Serruto, María Lazo‐Porras. **Writing – original draft**: Jemma Hawkins, Daniela Rossini‐Vilchez, Francisco Jose Tateishi‐Serruto, María Lazo‐Porras, Guillermo Almeida Huanca. **Writing – review & editing**: María Lazo‐Porras, Jemma Hawkins, Francisco Jose Tateishi‐Serruto, Daniela Rossini‐Vilchez, Guillermo Almeida Huanca, Miriam Lúcar‐Flores, Christopher Butler, María Sofía Cuba‐Fuentes, J Jaime Miranda, Antonio Bernabe‐Ortiz, Francisco Diez‐Canseco, Maria Kathia Cardenas, Graham Moore, Filipa Landeiro, Rafael A Calvo, William Whiteley.

## CONFLICT OF INTEREST STATEMENT

The authors declare no conflicts of interest. Author disclosures are available in the Supporting Information.

## CONSENT STATEMENT

All participants took part in the research voluntarily and provided written informed consent or assent. The procedures for this are detailed within the body of the manuscript.

## Supporting information




Supporting Information

Supporting Information

Supporting Information

Supporting Information

## Data Availability

The datasets generated or analyzed during this study will initially be available in anonymized format from the study investigators (M.L.P., J.H., S.C.F., and C.B.) upon reasonable request, in accordance with participants’ informed consent and institutional review board (IRB) requirements. No publicly accessible repository is currently used for data sharing, but this will be developed as part of the IMPACT Salud study and more information will be available on the study website (https://impact‐salud.org/es_pe) in due course.
