# Supplementary material for: The patient journey for people with dementia and their carers in Peru: From first symptoms to diagnosis and treatment
Source: Alzheimers Dement. 2026 Jul 9;22(7):e71581. doi: 10.1002/alz.71581 (PMC13351310; doi:10.1002/alz.71581)
Supplement: Supplementary file 1 — Supporting Information [file ALZ-22-e71581-s002.pdf]

Domain	Item	Description	Reported? (Yes/No/NA)	Page number
1. Research team and reflexivity	1	Who conducted the interviews or focus groups?	Yes	4
	2	What were the researcher's credentials?	Yes	4
	3	What was their occupation at the time of the study?	Yes	4
	4	Was the researcher male or female?	Yes	4
	5	What experience or training did the researcher have?	Yes	4
2. Relationship with participants	6	Was a relationship established prior to study commencement?	Yes	3
	7	What did participants know about the researcher?	Yes	4
	8	What characteristics were reported about the interviewer/facilitator?	Yes	4
3. Study design	9	What methodological approach was stated?	Yes	3
	10	How were participants selected? (e.g. purposive, convenience)	Yes	3
	11	How were participants approached?	Yes	3
	12	How many participants were in the study?	Yes	6
	13	Non-participation: How many people refused or dropped out?	Yes	3
	14	Setting of data collection: Where was the data collected?	Yes	4
	15	Presence of non-participants: Was anyone else present besides the participants and researchers?	Yes	4
	16	Description of sample: What are the important characteristics of the sample?	Yes	3
	17	Interview guide: Were questions, prompts, guides provided? Was it pilot tested?	Yes	4
	18	Repeat interviews: Were repeat interviews carried out?	N/A	-
	19	Audio/visual recording: Did the study use audio or visual recording?	Yes	4
	20	Field notes: Were field notes made during and/or after the interview?	N/A	-
	21	Duration: What was the duration of the interviews or focus groups?	Yes	5
	22	Data saturation: Was data saturation discussed?	N/A	-
	23	Transcripts returned: Were transcripts returned to participants for comment or correction?	N/A	-
4. Analysis and findings	24	Number of data coders: How many data coders were involved?	Yes	5
	25	Description of the coding tree: Did authors describe the coding tree?	Yes	5
	26	Derivation of themes: Were themes identified in advance or derived from the data?	Yes	5
	27	Software: What software, if any, was used to manage the data?	Yes	5
	28	Participant checking: Did participants provide feedback on the findings?	Yes	5
	29	Quotations presented: Were participant quotations presented to illustrate the themes/findings?	Yes	8-16
	30	Data and findings consistent: Was there consistency between data presented and the findings?	Yes	7-16
	31	Clarity of major themes: Were major themes clearly presented in the findings?	Yes	7-16
	32	Clarity of minor themes: Is there a description of diverse cases or discussion of minor themes?	Yes	7-16
