# Supplementary material for: The patient journey for people with dementia and their carers in Peru: From first symptoms to diagnosis and treatment
Source: Alzheimers Dement. 2026 Jul 9;22(7):e71581. doi: 10.1002/alz.71581 (PMC13351310; doi:10.1002/alz.71581)
Supplement: Supplementary file 2 — Supporting Information [file ALZ-22-e71581-s003.pdf]

Supplementary Material 3: Characteristics of the participants

Site	Type of interviewee	Sex of the PWD	Comorbidities of the PwD	Sex of the carer	Relationship with the PwD
Huancayo	Carer	F	Depression	F	Daughter
Huancayo	Carer	F	Arterial hypertension	F	Daughter
Huancayo	Carer	F	Fractures	F	Daughter
Huancayo	Carer	F	Depression, Hyperthyroidism	F	Daughter
Huancayo	PwD	F	Arterial hypertension	F	Daughter
Iquitos	Carer	M	Arterial hypertension	F	Ex-wife
Iquitos	Carer	F	Arterial hypertension	M	Grandson
Iquitos	Carer	M	Stroke, Arterial hypertension	F	Daughter
Iquitos	Carer	M	No specified	F	Daughter
Iquitos	PwD	M	Arterial hypertension	F	Daughter
Lima	Carer	M	Not specified	F	Wife
Lima	Carer	M	Diabetes, Depression	M	Son
Lima	Carer	F	Arterial hypertension	F	Daughter
Lima	PwD	M	Depression	F	Wife
Lima	PwD	F	Depression	F	Granddaughter
Lima	Carer	F	Parkinson	F	Daughter
Tumbes	Carer	F	Arterial hypertension	F	Daughter
Tumbes	Carer	F	Not specified	F	Daughter
Tumbes	Carer	F	Not specified	F	Daughter
Tumbes	Carer	M	Arterial hypertension, Diabetes	F	Paid carer
Tumbes	Carer	M	Diabetes	M	Son
Tumbes	Carer	M	Diabetes, Arterial hypertension	F	Ex-wife

Region	Type of interviewee	Sex of the participant	Speciality		Level of the health facility
Huancayo	Health care worker	F	Psychologist		Primary Level of Care
Huancayo	Health care worker	M	Family physician		Primary Level of Care
Huancayo	Health care worker	M	Psychologist		Secondary Level of Care
Huancayo	Health care worker	M	Doctor		Secondary Level of Care
Iquitos	Health care worker	F	Doctor		Primary Level of Carel
Iquitos	Health care worker	F	Doctor		Primary Level of Care
Iquitos	Health care worker	F	Psychologist		Primary Level of Care
Iquitos	Health care worker	F	Psychologist		Primary Level of Care
Lima	Health care worker	F	Doctor		Secondary Level of Care
Lima	Health care worker	F	Doctor		Secondary Level of Care
Lima	Health care worker	F	Doctor		Secondary Level of Care
Lima	Health care worker	M	Doctor		Secondary Level of Care
Lima	Health care worker	M	Psychologist		Primary Level of Care
Tumbes	Health care worker	F	Nurse		Primary Level of Care
Tumbes	Health care worker	F	Nurse		Primary Level of Care
Tumbes	Health care worker	F	Psychologist		Primary Level of Care
Tumbes	Health care worker	F	Psychologist		Primary Level of Care
