# Supplementary material for: The patient journey for people with dementia and their carers in Peru: From first symptoms to diagnosis and treatment
Source: Alzheimers Dement. 2026 Jul 9;22(7):e71581. doi: 10.1002/alz.71581 (PMC13351310; doi:10.1002/alz.71581)
Supplement: Supplementary file 3 — Supporting Information [file ALZ-22-e71581-s004.pdf]

PREDIAGNOSIS (patient and carer)

Dementia symptoms

Care for other types of comorbidities

Health center prevention activities

Depression
Memory loss
Aggressiveness
Confusion

Health Centres (HC)
Community Mental Health Centres (CMHC)
Hospital
Hospitals in other cities
Emergency in hospitals

Referring to:
- Geriatrics
- Psychiatry
- Neurology
- ...

Other illnesses

Care for other types of comorbidities

Health center prevention activities

Hypertension
Accident
Hearing loss
Stroke
Loss of appetite
Insomnia and headache

Health Centres (HC)
Community Mental Health Centres (CMHC)
Hospital
Hospitals in other cities
Emergency in hospitals

Referring to:
- Geriatrics
- Psychology
- Neurology
- ...

Symptoms of dementia and other diseases

Care for other types of comorbidities

Health center prevention activities

Aggressiveness
Hypertension
Hearing loss
Stress
Memory loss
Depression
Accident
Loss of appetite
Insomnia and headache

Health Centres (HC)
Community Mental Health Centres (CMHC)
Hospital
Hospitals in other cities
Emergency in hospitals

Referring to:
- Geriatrics
- Psychology
- Neurology
- ...

IMPACT SALUD
FUNDED BY NIHR | National Institute for Health and Care Research
CRONICAS
CENTRO DE EXCELENCIA EN ENFERMEDADES CRÓNICAS

DIAGNOSIS (patient and carer)

Health Centres

Admission → Triage → Consultation (Private laboratories) → Possible referral to hospital or CMHC (Private clinic)

Hospital

Administrative staff → Technical staff → Neurologist, Psychologist, Geriatrician, Psychiatrist (Private clinic; Private laboratories) →

Comprehensive geriatric assessment

- Speciality 1
- Speciality 2
- Speciality 3
- **Diagnosis**

→ **Test**

- laboratory
- psychological
- images

They may be referred to the CMHC to continue their treatment

Community Mental Health Centres (CMHC)

*They request imaging and/or care in the hospital's neurology department.

Nurse →

P.A.I/E.I

Nurse
Psychologist
Doctor Psychiatrist
Social worker
Technician

(Private laboratories) → **Diagnosis** (Private clinic)

Diagnosis outside the health system → Healthcare workers outside hospital public

"A person may receive multiple diagnoses from different doctors."

Continuity of treatment

Hospital

The treatment can be managed by a specialist

Neurologist / Geriatrician / Psychologist / Psychiatrist

interconsultation

non-pharmacological treatments

- Regular consultations
  Seen every four months
- Regular therapy appointments
  Psychotherapy: psychologist
  Physical therapy
  Cognitive stimulation therapy

Pharmacological treatments:

- Public pharmacies
  Private pharmacies

When necessary, home visits are made by the social worker. → referrals CMHC

Community Mental Health Centres (CMHC)

Dementia care package

Pharmacological treatments

- Public pharmacies
  Private pharmacies

a Psychiatry / Family doctor

- Monthly consultation

*They request images and/or attention from the neurology department at the hospital.

b Therapy/Workshops

- Behavioural psychotherapy (also applicable to caregivers)
- Psychoeducational therapies (sociability)
- Occupational therapies (functional independence)

c Home visits

- Adherence to pills

*It depends on the availability of resources at the CMHC

d Telemonitoreo/ Telemedicina

*Only in cases where they can not go to the CMHC, for adherence to treatment

Health Centres (HC)

Recommendations from healthcare workers

- Walk
- Take sleeping pills
- Don't sleep too much
- Don't contradict him
- Talk to friends
- Diet

IMPACT SALUD
FUNDED BY NIHR | National Institute for Health and Care Research
CRONICAS
CENTRO DE EXCELENCIA EN ENFERMEDADES CRÓNICAS
